# Design of Microcapsules for Self-Healing Concrete Based on Fracture Modeling of RVE and UC with PBC Using XFEM and CS Technique

**DOI:** 10.3390/ma19132878

**Published:** 2026-07-06

**Authors:** John Hanna, Martin Drieschner

**Affiliations:** Chair of Structural Analysis and Dynamics, Brandenburg University of Technology Cottbus-Senftenberg, Konrad-Wachsmann-Allee 2, 03046 Cottbus, Germany; martin.drieschner@b-tu.de

**Keywords:** self-healing concrete (SHC), microcapsules design, fracture modeling, representative volume element (RVE), unit cell (UC), periodic boundary conditions (PBC), eXtended finite element method (XFEM), cohesive surface (CS), volume fraction (*V_f_*), interfacial cohesive properties, microcapsule core–shell thickness ratio

## Abstract

The fundamental issue in designing encapsulation-based self-healing concrete structures is the design of microcapsules. However there are few studies in the literature on this topic; not only is the fracture of microcapsules crucial for releasing the healing agent to heal fractures in the concrete matrix, but also the amount of the healing agent and expected crack widths. Therefore, in this paper, a novel design method of dimensioning microcapsules for encapsulation-based self-healing concrete (SHC) with consideration for a sufficient volume of healing agent to heal a specific crack width is developed. It is based on the configuration of the representative volume element (RVE) and the unit cell (UC), and associates them with the volume fraction (*V_f_*) and the crack width as variables with applied periodic boundary conditions (PBCs). It is also validated through numerical fracture modeling using the eXtended Finite Element Method (XFEM), and cohesive surface (CS) technique. Effects of interfacial cohesive properties, the microcapsule size, and volume fraction on the load carrying capacity and the crack pattern are investigated numerically. The obtained results are in good agreement with the literature. The developed design method can serve as a valuable tool for obtaining a preliminary design of microcapsules for SHC.

## 1. Introduction

**Figure 1:**
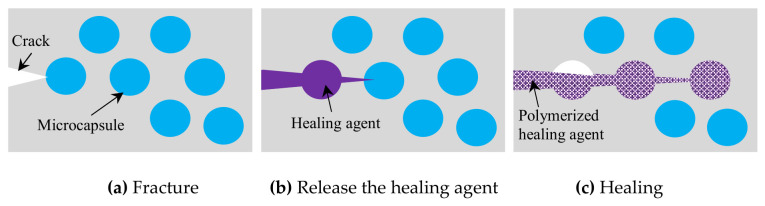
Encapsulation-based self-healing using microcapsules.

The ability of encapsulation-based self-healing concrete (SHC) to automatically repair cracks without the need for human intervention has made it a promising technique among others for autonomous self-healing concrete. This ability can prolong the lifespan of concrete structures and lower their maintenance costs [1,2]. It has great prospects for infrastructures because as the propagating cracks strike the capsule shell, the healing agents are released on their own to cure the fractures around the damaged zone; Figure 1. Various healing agents, such as polymer, bacteria, sodium silicate, and various capsule shell materials, e.g., glass, ceramic, gelatin, silicon, expanded clay, cementitious, and polymers have been developed and tested, aiming to extend the life of the healing agent, accelerate the reaction after cracking and improve the healing capability. Also several shapes and sizes of capsules such as vascular, capillary, spherical, and tubular/cylindrical, are investigated [2,3,4]. A recent study developed a capsule-based self-healing system suitable for concrete in CO_2_-rich environments; in sewer systems due to microbial activities, there has always been a degradable factor, which accelerates the corrosion inside concrete sewer pipes. The capsules, utilizing water treatment sludge, calcium hydroxide, and expansive polymers, significantly restore water tightness and compressive strength through the formation of calcite crystals [5].

The fracture of the capsules, the bonding between the capsules and the concrete matrix, the healing efficiency, and the fracture process have all been the subject of numerous laboratory investigations and experiments, for example [6,7]. Also several computational modeling techniques based on the cohesive zone model (CZM) have been used in SHC to investigate the interaction between capsules and concrete matrix, such as the zero thickness cohesive element approach [8] and the extended finite element method (XFEM) with the cohesive surface (CS) technique, which showed more accuracy [9,10]. The zero thickness cohesive element approach has been widely used for fracture modeling in the literature, although it has a number of important shortcomings, introducing artificial compliance and mesh dependency [11]. The extended finite element method (XFEM) has proven itself to be a promising, flexible, and effective discrete crack technique that allows fracture propagation without re-meshing. Furthermore, it has been demonstrated to model fracture in concrete materials with excellent accuracy [12], and it is already incorporated into commercial software such as ABAQUS [13]. Moreover, computational modeling has demonstrated benefits in simulating physical phenomena including fracture energy, healing patterns, and capsular clustering that are hard and difficult to study experimentally [14,15,16].

Despite extensive research into self-healing mechanisms, there remains a notable lack of experimental and modeling studies addressing the specific design parameters of these systems. To the author’s best knowledge, the optimization of capsule size—the primary component of such techniques—has not been sufficiently explored. While the existing literature has addressed capsule development, the focus has predominantly remained on material characterization (e.g., [17,18]) rather than geometric design. The determination of capsule size is a critical primary issue in the design of self-healing systems, as the requisite volume of the healing agent is intrinsically linked to capsule dimensions, volume fraction (*V_f_*), and the projected crack width. The key novelty of this study lies in the development of a systematic design methodology for dimensioning capsules to ensure a sufficient supply of healing agent for a target crack width. The proposed method utilizes the configurations of the unit cell (UC) and representative volume element (RVE), integrated with periodic boundary conditions (PBCs), while treating *V_f_* and crack width as the fundamental variables. To establish a comprehensive framework, the following subsections summarize the essential concepts of microstructural configuration modeling.

### 1.1. Representative Volume Element (RVE)

The literature provides several definitions for the representative volume element (RVE), all of which relate to identifying an adequate volume that represents the microstructural geometry and its effective properties. According to one convention, an RVE must serve as a statistically representative sample of the microstructure, encompassing a complete sampling of all possible microstructural configurations [19]. This definition leads to a considerably large RVE for a nonuniform microstructure which is computationally inefficient. An alternative definition characterizes the RVE as the smallest microstructure capable of sufficiently representing the macroscopic properties of interest, such as the constitutive relationship between stress and strain. While this results in a more computationally efficient size, an RVE satisfying this criterion may not always capture the full distribution of internal microfields. In the context of the present numerical simulations, the RVE is defined as the smallest material volume that accurately represents the structural integrity of the material.

### 1.2. Unit Cell (UC)

The unit cell (UC) is defined as a discrete portion of a material at a lower length scale that can reproduce the entire material domain through appropriate symmetry transformations. When correctly defined, the UC and its translated images fill the entire space without gaps or overlaps. The existence of a UC implies a degree of regularity in the material architecture at the lower scale, which ensures homogeneity at the macroscopic level. While macroscopic homogeneity can also arise from a random distribution at the lower scale based on statistical considerations, the application of a UC typically serves as either a realistic representation of periodic architecture or an idealization of an otherwise random structure. As noted in [20], if the inherent randomness of the lower-scale architecture must be explicitly modeled, a statistical RVE must be employed rather than an idealized UC.

Generally spherical capsules have less influence on the mechanical properties as their shape reduces the stress concentrations around the void left from empty capsules [2,3]. Therefore, the developed design method in this paper focused on spherical microcapsules. However the same principals used in this design method can be applied for any shapes of capsules. The healing mechanism, which occurs by releasing the healing agent once the capsules are cracked, depends critically on the fracture of the capsules. As a result, the developed design method is also validated through numerical fracture modeling using extended finite element method (XFEM), and cohesive surface (CS) technique for the previously mentioned advantages. In this validation, the effects of microcapsule size and the volume fraction (*V_f_*) on the load carrying capacity and the possibility of debonding or fracture of the microcapsules from the concrete matrix are numerically investigated. 2D numerical fracture modeling is performed for the obtained SHC design samples including the microcapsules loaded under uniaxial tension. XFEM is used for modeling the concrete matrix and the microcapsule shell, while the interaction between them is modeled by CS with PBCs applied. The potential use of the developed design method could act as a helpful tool to obtain a preliminary design of microcapsules for self-healing concrete without any complications, especially for costly detailed modeling. 

## 2. Proposed Design Method of Microcapsules/Design Sample

**Figure 2:**
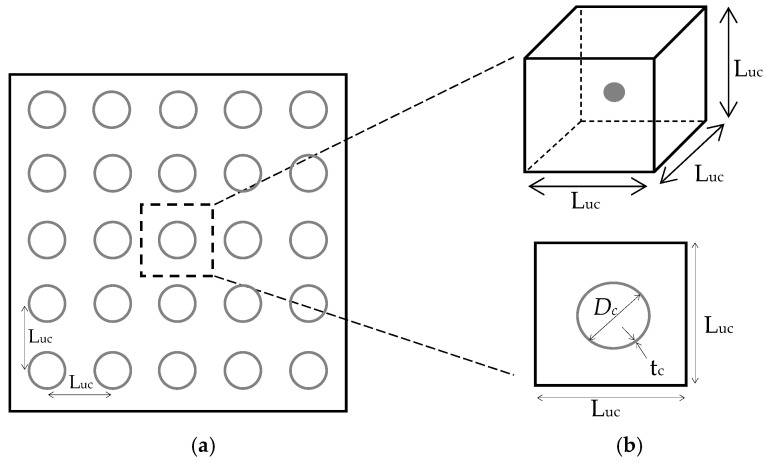
Geometry dimensions of (**a**) RVE and (**b**) UC of the proposed design method of microcapsules.

It is assumed that the spherical microcapsules are uniformly distributed in an infinite concrete matrix domain. Therefore, the material can be represented using a representative volume element (RVE) with multiple uniform distributed microcapsules, as illustrated in Figure 2a. Due to the regularity in the distribution of microcapsules, the material also can be represented using a unit cell (UC) with a single microcapsule as illustrated in Figure 2b. The UC for this hypothesis is a cube, and includes a single microcapsule at its centroid. The three dimensions of the cube have the same length (L_uc_) and at the same time it is equal to the interdistance between the periodic distributed microcapsules. Hence L_uc_ can be defined as the dimension of the SHC design sample. The microcapsule dimensions are represented by the outer diameter (*D_c_*) and the wall shell thickness (t_c_). In order to model this proposed design method of microcapsules, the periodic boundary conditions (PBCs) should be applied on the outer edges of the SHC design sample, which is basically the UC, to obtain a proper mechanical response of such infinite SHC domains represented by RVE. Although the hypothesis of the uniform distribution microcapsules is somewhat far from the real random distribution of microcapsules, it is suitable to provide a simple method to design the microcapsule based on the definition of RVE and UC. However, the effects of microcapsule clustering, which could be generated from the real random distribution of microcapsules, are studied computationally on the fracture mechanism based on a simple developed approach in another study [14].

### 2.1. Virtual Domain of the Unit Cell

The volume fraction (*V_f_*) is a parameter which represents the volume ratio of the microcapsule to the unit cell (UC) of the concrete matrix—see Equation (3). The volume of the microcapsule is calculated based on its outer diameter (D_c_) as shown in Equation (1) and the volume of the unit cell shown in Equation (2).


(1) $$V_{c}=\frac{\pi D_{c}^{3}}{6}$$

(2) $$V_{m}=L_{uc}^{3}$$

(3) $$V_{f}=\frac{V_{c}}{V_{m}}$$

Substituting Equations (1) and (2) into Equation (3), *V_f_* could be expressed as follows:


(4) $$V_{f}\;=\;\frac{\pi D_{c}^{3}}{6\;L_{uc}^{3}}$$

From Equation (4) the following equation of L_uc_ could be obtained:


(5) $$L_{uc}\;=D_{c}\;\sqrt[{3}]{\frac{\pi }{6\;V_{f}}}$$

This equation relates the dimension of UC (L_uc_) which is equal to the distance between microcapsules to the microcapsule size represented by its diameter *D_c_*. *V_f_* can be estimated based on the expected crack width and the required volume of healing agent required to heal that crack.

### 2.2. Expected Crack Width of the Unit Cell

It is assumed that only one crack will be generated inside the design sample and break the microcapsule. Therefore the healing agent is encapsulated in a microcapsule, which, placed inside the SHC design sample, must be sufficient to heal the crack surface around it. Taking into account the roughness of the crack surface and the possibility that the healing agent will not be completely released from the fractured microcapsule and there will be some healing agent remaining in it. So, it is assumed that the volume of the released healing agent is half of the microcapsule volume. Assuming also that the crack width (t_cr_) is constant along the crack path, the volume of the healed crack (V_cr_) and L_uc_ can be obtained as follows:


(6) $$V_{cr}=\frac{V_{c}}{2}={\left(L_{uc}-\frac{D_{c}}{2}\right)}^{2}t_{cr}$$

(7) $$L_{uc}=\sqrt{\frac{V_{c}}{2t_{cr}}}+\frac{D_{c}}{2}$$

**Figure 3:**
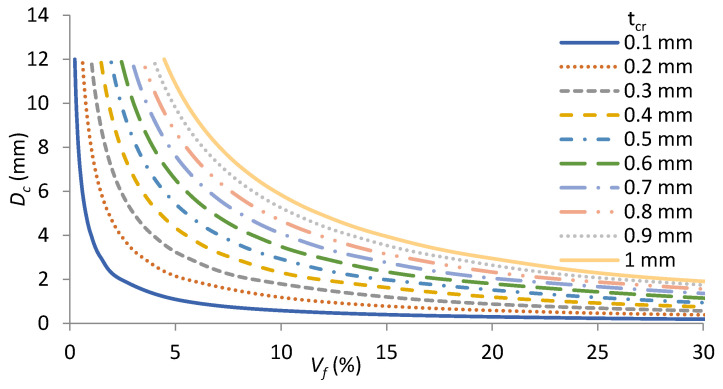
Design curves of microcapsules.

Design curves of microcapsules can be developed based on Equations (4) and (7) depending on the capsule diameter, volume fraction, and the expected crack width which can be healed, as shown in Figure 3. It can be seen easily from these curves that a range of volume fraction between 5% and 25% for a range of microcapsule diameters between 2 mm and 12 mm will be able to heal the crack width ranging from 0.1 mm to 1 mm.

## 3. Modeling Framework

**Figure 4:**
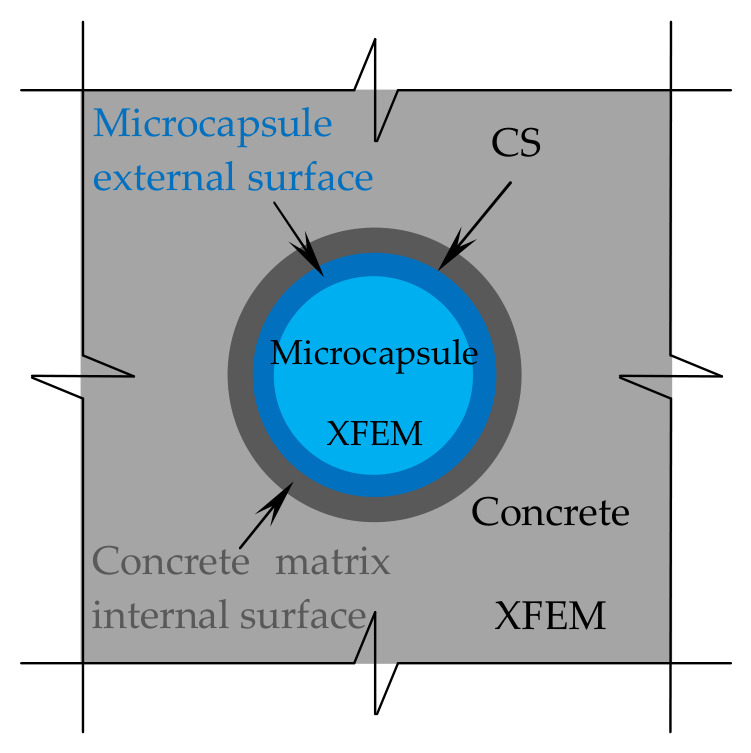
Zoom view of the modeling techniques.

In this study, the simulations of the proposed microcapsule design method as discussed in the previous section are performed to study the effects of microcapsule size, volume fraction (*V_f_*), and interfacial fracture properties between the microcapsule shell and the concrete matrix on the load carrying capacity and the possibility of the microcapsule fracture or debonding from the concrete matrix. The concrete matrix, microcapsule shell, and interaction between them compose the three phases of the specimens that are modeled. Periodic boundary conditions (PBCs) are applied to the outer edges of the SHC design sample using two computational techniques to carry out the simulations. The fracture propagation in both the concrete and the microcapsule has been modeled using the eXtended Finite Element Method (XFEM). The interaction between these two components is modeled by the cohesive surface (CS) technique, as shown in Figure 4. A traction–separation law serves as the basis of damage modeling for both computational techniques.

### 3.1. Extended Finite Element Method (XFEM)

**Figure 5:**
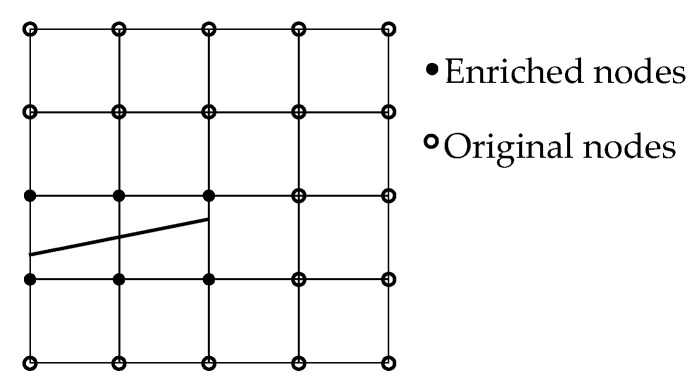
Crack with enriched elements.

The concept of local partition of unity serves as the basis for the extended finite element method (XFEM), which could be considered as an extension of the common finite element method. As a result, the cracks can be simply integrated into a finite element mesh and represented as local enrichment functions, avoiding the need for remeshing throughout the simulation [21]—see Figure 5. Cracks are represented by enrichment functions that are integrated into the usual displacement interpolation function, Equation (8) [22]. The standard nodes are not displayed, while the enriched nodes are indicated by black dots, and the crack-tip enriched node by white dots. XFEM is a highly promising approach for fracture modeling, as it has already been incorporated into commercial software like Abaqus [13,23]. More detailed description of this modeling method can be found in [21]. The concrete matrix and microcapsule shell cracks inside Abaqus are modeled by XFEM, which is defined in the interaction module.


(8) $$u=\sum_{I=1}^{N}N_{I}\left(x\right)\left[u_{I}+H\left(x\right)a_{I}+\sum_{\alpha =1}^{4}F_{\alpha }(x)b_{I}^{\alpha }\right]$$

Shape functions are represented by *N_I_*(x), nodal displacement vectors are represented by *u_I_*, jump functions, like Heaviside functions, are represented by *H*(x), nodal vectors of the enriched degree of freedom are represented by *a_I_* and *b_I_^α^*, and crack-tip functions are represented by *F_α_*(x). The first term on the right affects every node in the model, whereas the second term only affects nodes whose shape function support crosses the inner area of the crack. The third term is applied only to nodes whose form function support extends to the crack tip. This third term is called the “crack tip enrichment” and contains the information of the analytical solution in linear elastic fracture mechanics. However, concrete materials cannot be well handled by linear elastic fracture mechanics. For this reason, in this study the final term on the right-hand side has been eliminated. Consequently, as Figure 5 illustrates that the crack tip will be at the element edge rather than inside the element where it cannot be stopped. XFEM has few drawbacks despite its many benefits for fracture modeling [23]. Two main limitations need to be taken into account. The first one is related to fracture propagation close to the element edge. Allowing numerous cracks to initiate in the same enrichment zone is the second. However, to get around this restriction, there is a setting in the Abaqus input file; see [9,23] for more detailed information.

### 3.2. Cohesive Surface Technique (CS)

The cohesive surface (CS) is a modeling technique that uses the traction–separation response to simulate a zero-thickness contact interface between two surfaces [9,23]. The interaction between the microcapsule and the concrete matrix is modeled in this work using the CS technique. Pure master–slave roles are used to model it in the contact formulation and it is characterized as a cohesive surface interaction property in Abaqus. Within this work, the concrete matrix’s interior surface is referred to as a master surface and the microcapsule’s outside surface as a slave surface.

### 3.3. Cohesive Zone Model (CZM)

**Figure 6:**
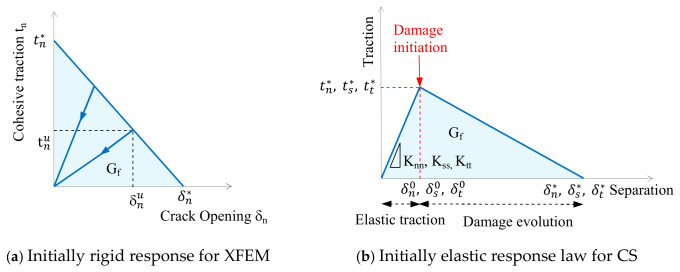
Traction–separation law.

The cohesive zone model (CZM) is used across the crack surface that links the displacement jump, which is defined by the separation vector, to the cohesive traction conveyed by the discontinuity surface. It uses a traction–separation law to model the failure mechanism of cracks. Figure 6 shows the initially rigid CZM for XFEM, and the initially elastic CZM for CS, which is represented by the linear zone (linear elastic traction) followed by the softening zone (damage evolution). Damage initiation and damage evolution are the two criteria that Abaqus uses to describe both models. A cracked element’s elastic behavior can be represented as an elastic constitutive matrix that links shear and normal stresses with shear and normal separations. The nominal traction stress vector (t) in the 3D modeling is made up of three components.

t_n_, t_s_, and t_t_, represent the normal and shear tractions in two directions, respectively. The corresponding separations are denoted by δ_n_, δ_s_, and δ_t_. The elastic behavior can then be written as:


(9) $$t=\left\{\begin{array}{c} t_{n}\\ t_{s}\\ t_{t} \end{array}\right\}=\left[\begin{array}{ccc} K_{nn} & 0 & 0\\ 0 & K_{ss} & 0\\ 0 & 0 & K_{tt} \end{array}\right]\left\{\begin{array}{c} \delta_{n}\\ \delta_{s}\\ \delta_{t} \end{array}\right\}=K\delta$$

The elastic response is governed by the penalty stiffnesses, K_nn_, K_ss_, and K_tt_, which are calculated as functions of the two neighboring material stiffnesses [24]. These values have been taken to be 1 × 10^6^ MPa/mm in this paper, and they have no effect on the overall stiffness of the specimen [25]. Furthermore, it is assumed that the shear and normal penalty stiffnesses are decoupled, meaning that shear forces are not produced by the interface’s pure normal opening force and vice versa [9]. By default, the elastic zone is assumed to be linear. For the softening zone, there are several damage evolution laws available, such as the linear and nonlinear traction–separation law [23]. The softening in this research is specified as linear, implying the use of a bilinear transcription–separation law—see Figure 6b. Although the traction–separation behavior is defined inside the material properties for the XFEM, it is specified as part of the interaction properties for the CS.

#### 3.3.1. Damage Initiation

Numerous damage initiation criteria have been successfully included into software such as Abaqus and are described in the literature. These criteria include, for example, the maximum nominal stress criterion, quadratic stress criterion, maximum separation criterion, and quadratic separation criterion. When the maximum stress is more than the material’s maximum strength, damage will start in the case of the stress criteria. In this work, the maximum principal stress damage criteria is applied to the XFEM. Consequently, if the maximal primary stress obtained by its integration points satisfies the condition of Equation (10), a crack may develop. Nevertheless, the CS technique uses the maximum nominal stress damage criterion, which implies that a separation can occur when the greatest nominal traction satisfies Equation (11). In this context, a more thorough explanation may be found in [23].


(10) $$max\left\{0,\frac{\sigma_{maxps}}{\sigma^{*}}\right\}\geq 1$$

(11) $$max\left\{\frac{\langle t_{n}\rangle }{t_{n}^{*}},\frac{t_{s}}{t_{n}^{*}},\frac{t_{t}}{t_{t}^{*}}\right\}=1$$

σ* is the maximum strength of the material, and σ_maxps_ is the maximum primary stress that can be calculated. The letters n, s, and t stand for the normal, shear, and tangential components of the interfacial tractions, respectively. * stands for the maximum interfacial tractions.

#### 3.3.2. Damage Evolution

The damage evolution characterizes the cohesive stiffness deterioration by specifying the softening portion of the traction–separation law. The scalar damage variable (D) has an initial value of 0 and increases to 1 throughout the loading process. It is necessary to specify in Abaqus the maximum displacement or the fracture energy, which is the area under the traction–separation law’s curve [23]. The damage evolution with linear softening is defined in this work using the fracture energy. An effective separation is defined using the approach described in [26] to characterize the development of damage when both normal and shear separations exist across the interface:


(12) $$\delta_{m}=\sqrt{{\langle \delta_{n}\rangle }^{2}+{\delta_{s}}^{2}+{\delta_{t}}^{2}}$$

(13) $$D=\frac{\delta_{m}^{*}}{\delta_{m}^{u}}\left(\frac{\delta_{m}^{u}-\delta_{m}^{0}}{\delta_{m}^{*}-\delta_{m}^{0}}\right)$$

(14) $$t_{n}=\{\begin{array}{l} \left(1-D\right){\bar{t}}_{n}\;\;\;\;\;\;\;\;\;\;\;if\;t_{n}\geq 0\\ {\bar{t}}_{n}\;\;\;\;\;\;\;\;\;\;\;\;\;\;\;\;\;\;\;\;\;\;\;\;\;if\;t_{n}<0\;\left(compression\right) \end{array}$$

(15) $$t_{s}=\left(1-D\right){\bar{t}}_{s}$$

(16) $$t_{t}=\left(1-D\right){\bar{t}}_{t}$$

The directions of the interfacial separations’ normal, shear, and tangential components are, respectively, $\delta_{n},\;\delta_{s},\;\mathrm{and}\;\delta_{t}$. While $\delta_{m}^{*}$ is the maximal effective separation during loading, $\delta_{m}$ is the effective separation. The effective separation at the initial stage of the damage process is $\delta_{m}^{0}$, whereas the effective separation right before unloading is $\delta_{m}^{u}$. This is in contrast to the elastic traction–separation behavior, which states that ${\bar{t}}_{n},\;{\bar{t}}_{s},\;\mathrm{and}\;{\bar{t}}_{t}$ are the contact traction components for current separations without damage. Utilizing Macaulay brackets ⟨⟩ helps prevent compressive damage.

The energy dissipated to produce a totally separated pair of surfaces is called the interface fracture energy (G_f_), and it is represented by the region beneath the curves in Figure 6. A cohesive interface for healed cracks may have a mixed-mode propagation response depending on where the interface is placed in relation to the applied stress. This mixed mode includes a range of energies linked to the ability of debonding in both the normal (n) and parallel (s, t) directions to the interface. The formula given in [26] is then used to calculate the maximum fracture separation:


(17) $$\delta_{n}^{*}=\frac{2G_{n}^{*}}{t_{n}^{0}}$$

(18) $$\delta_{s}^{*}=\frac{2G_{s}^{*}}{t_{s}^{0}}$$

(19) $$\delta_{t}^{*}=\frac{2G_{t}^{*}}{t_{t}^{0}}$$

The interaction between the energies for each mode, n, s, and t, is assumed in this study to satisfy the power law fracture requirement as described in [26]:

(20) $${\left\{\frac{G_{n}}{G_{n}^{*}}\right\}}^{\alpha }+{\left\{\frac{G_{s}}{G_{s}^{*}}\right\}}^{\alpha }+{\left\{\frac{G_{t}}{G_{t}^{*}}\right\}}^{\alpha }=1$$

where G_n_, G_S_, and G_t_ are the energy release rates obtained from the traction and normal, shear, and tangential displacements during interface opening, and power α is a cohesive property parameter that characterizes the interaction between modes. For each direction, the critical interface toughness is represented by the properties $G_{n}^{*},\;\;G_{s}^{*},\;{\;and\;G}_{t}^{*}$. It is assumed in this study that the critical fracture toughness is constant in all directions. Additionally, a value of α = 1 has been used [26] to take into account the impact of this parameter on the response.

### 3.4. Periodic Boundary Conditions (PBCs)

**Figure 7:**
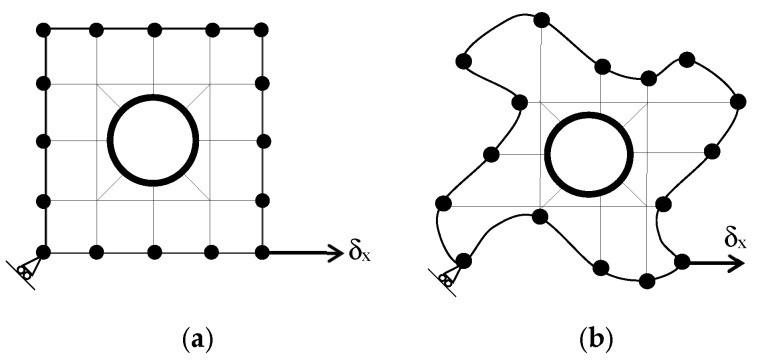
Typical periodic boundary conditions (**a**) and the associated deformation shape (**b**).

Periodic boundary conditions are usually used in micromechanics for the study of heterogeneous media. The periodic boundary condition (PBC) stipulates that opposite pairs of edges or surfaces on the boundary of an RVE should deform identically under a given loading history. The PBC basically prevents the constraint of enforcing edges to remain planar after deformation [27], as illustrated in Figure 7 for 2D RVE.

**Figure 8:**
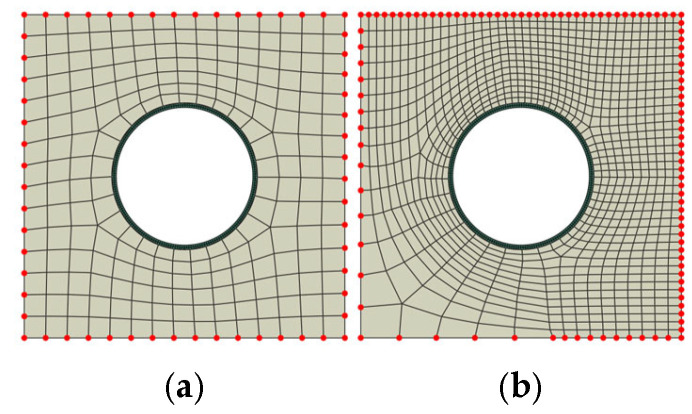
Periodic mesh (**a**) versus non-periodic mesh (**b**).

The implementation of PBCs within FEM is classified according to the type of mesh discretization. Generally, the mesh discretization could be a periodic or non-periodic mesh. In the case of the periodic mesh, the number of nodes on one edge is equal to the number of nodes on the other opposite edge—see Figure 8a. In the other case with the non-periodic mesh, the number of nodes on one edge is not equal to the number of nodes on the other opposite edge—see Figure 8b. The implementation of PBCs on the non-periodic mesh has some complications and is out of the scope of this work. Due to the proposed design method of microcapsules, and based on the unit cell, which does not generate any complication with mesh discretization, and the periodic mesh which can be generated easily, in this paper, the implementation of PBCs focused only on the periodic mesh.

**Figure 9:**
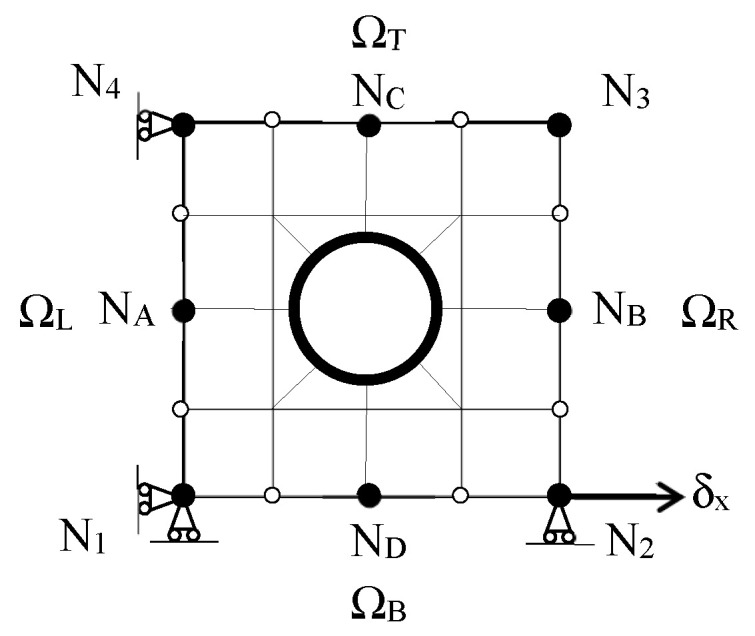
Implementation of PBC within periodic FEM mesh.

In order to implement PBC on a periodic mesh, consider a 2D RVE which is pinned at one of the corner nodes N_1_ to prevent rigid body motion, as illustrated in Figure 9. Every edge node should be linked kinematically on opposite edges. The principle is that the displacement of node b must be equal to the displacement of node a and so on, as expressed in Equations (21)–(24).

(21) $$U_{\left(x,y\right)}^{N_{B}}=U_{\left(x,y\right)}^{N_{A}}\;\;\;\;hence\;\;\;\;\;U_{\left(x,y\right)}^{N_{B}}-U_{\left(x,y\right)}^{N_{A}}=0$$

(22) $$U_{\left(x,y\right)}^{N_{C}}=U_{\left(x,y\right)}^{N_{D}}\;\;\;\;hence\;\;\;\;U_{\left(x,y\right)}^{N_{C}}-U_{\left(x,y\right)}^{N_{D}}=0\;$$

(23) $$U_{\left(x,y\right)}^{N_{2}}=U_{\left(x,y\right)}^{N_{1}}\;\;\;\;hence\;\;\;\;\;U_{\left(x,y\right)}^{N_{2}}-U_{\left(x,y\right)}^{N_{1}}=0$$

(24) $$U_{\left(x,y\right)}^{N_{4}}=U_{\left(x,y\right)}^{N_{1}}\;\;\;\;hence\;\;\;\;U_{\left(x,y\right)}^{N_{4}}-U_{\left(x,y\right)}^{N_{1}}=0\;$$

The boundary condition can be expressed as mathematical equations (canonical equations) where the displacements of the internal edge nodes are kinematically tied to the homogeneous displacements of the corner retained nodes. Note that N_3_ is not considered a retained node as its deformation is influenced by the deformations of nodes N_2_ and N_4_; this is why it is called a slave or a dummy node. In order to enforce an axial deformation ($\delta_{x}$), the displacements of the corner retained nodes must be linked kinematically to the edge internal nodes. Because the loads are applied on the corner nodes, the previous displacement boundary equations (canonical equations) can be rearranged as follows:


(25) $$U_{\left(x,y\right)}^{N_{B}}-\;U_{\left(x,y\right)}^{N_{A}}-\;U_{\left(x,y\right)}^{N_{2}}+\;U_{\left(x,y\right)}^{N_{1}}=0$$

(26) $$U_{\left(x,y\right)}^{N_{C}}-U_{\left(x,y\right)}^{N_{D}}-U_{\left(x,y\right)}^{N_{4}}+U_{\left(x,y\right)}^{N_{1}}=0\;$$

**Figure 10:**
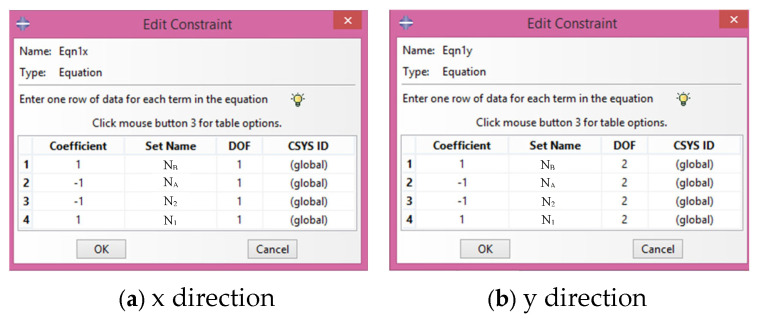
Constraint equations of PBCs in Abaqus interaction module.

The PBC is imposed by applying the above canonical equations on the boundary nodes. So, if node 2 moves a certain distance, every other thing in the model will have a connection; there is a connectivity between them and so the system will respond accordingly. The canonical equations are introduced into Abaqus using a multifunction constraint equation in the interaction module. Due to the simulation here in 2D, the canonical equations need to be defined in two degrees of freedom (DOF); x and y directions at every edge node as shown in Figure 10 respectively for nodes N_B_ and N_A_. The same principle is also applied on the top and bottom edges of RVE; for example, N_C_ and N_D_ linked kinematically to the corner nodes N_1_ and N_4_.

## 4. Numerical Modeling

**Figure 11:**
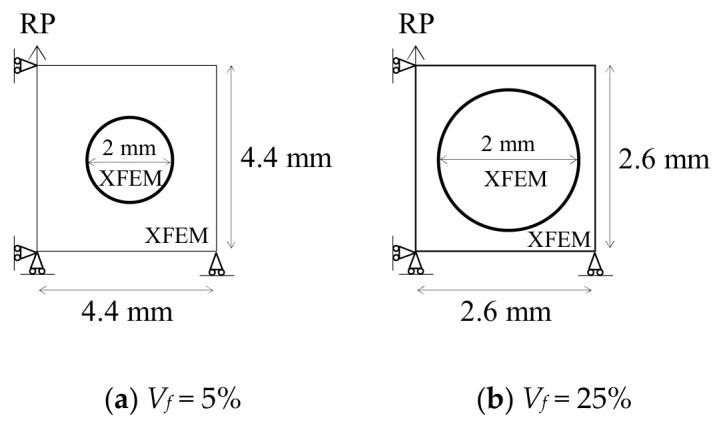
Geometry dimensions of design samples and modeling techniques.

2D SHC design samples, which are basically RVE body-centered unit cell samples with one microcapsule and volume fraction (*V_f_* = 5% and *V_f_* = 25%), are loaded under uniaxial tension. The outer diameter of the microcapsule is 2 mm with four values of core–shell ratio: 1:1, 5:1, 10:1, and 15:1. The microcapsule with the thinnest shell is the one with the biggest core–shell ratio (15:1), and vice versa. The dimensions of the design samples are calculated according to Equation (5); L_uc_ = 4.4 mm for *V_f_* = 5% while L_uc_ = 2.6 mm for *V_f_* = 25%. Since in this study it is important to investigate how the crack will initiate, there is no need for a preexisting crack. Figure 11 displays the overall geometric dimensions of both specimens with the boundary conditions. The PBCs have been implemented along the four sides of the design sample using equation constraints to connect each node with its corresponding node on the other side. Vertical displacement 0.05 mm is applied to the reference point (RP). The Abaqus/Static simulation was run on the assumption of plane stress conditions. Table 1 shows the used material properties, which are characterized according to [9,17,28,29,30,31]. Young’s modulus (E), Poisson’s ratio (ν), maximum tensile strength (σ*), and fracture energy (G_f_) are used to represent their properties. The cohesive surface that represents the interface between the microcapsule and the concrete matrix is assumed to have equal normal and shear fracture properties due to a lack of experimental verification in the literature.

### 4.1. Mesh Convergence

**Figure 12:**
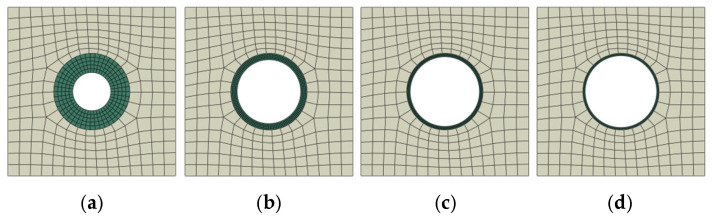
The mesh discretization for *V_f_* = 5% with different microcapsule core–shell ratios, (**a**) 1:1 (**b**) 5:1 (**c**) 10:1 (**d**) 15:1.

**Figure 13:**
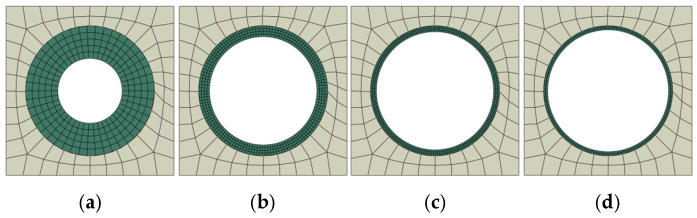
The mesh discretization for *V_f_* = 25% with different microcapsule core–shell ratios, (**a**) 1:1 (**b**) 5:1 (**c**) 10:1 (**d**) 15:1.

Several preliminary calculations, similar to those performed in [10,14], have been carried out to investigate the mesh convergence as a means of balancing accuracy with computing effort. The design samples are meshed with quadrilateral elements (Q4) with full integration. In order to have a periodic mesh to facilitate the application of PBC, the average mesh element size of the design sample is 0.44 mm; the number of elements for each side is fixed at 15 for *V_f_* = 5%, as shown in Figure 12. For *V_f_* = 25% the average mesh elements size of the design sample is 0.26 mm, and the number of elements for each side is fixed at 10, as shown in Figure 13. The number of circumferential elements around the microcapsule opening is fixed at 25 for both cases. The average mesh element size for microcapsule core–shell ratio 1:1 is 0.1 mm with 5 elements through its thickness, for microcapsule core–shell ratio 5:1 is 0.0417 mm with 4 elements through its thickness, for microcapsule core–shell ratio 10:1 is 0.0303 mm with 3 elements through its thickness, and for microcapsule core–shell ratio 15:1 is 0.03125 mm with 2 elements through its thickness.

### 4.2. Parametric Studies

The effects of the interfacial transition zone (itz) between the microcapsule shell and the concrete matrix are investigated through parametric studies of various interfacial fracture properties. Four different microcapsule core–shell ratios—1:1, 5:1, 10:1, and 15:1—are studied, as shown in Figure 12 and Figure 13 for *V_f_* = 5% and 25%, respectively, regarding load carrying capacity and crack patterns. The interfacial fracture properties are represented by two parameters that define the cohesive surface between the concrete matrix and the microcapsule shell: the maximum interfacial tensile strength σ*, which is known also as the bonding strength, and the interfacial fracture energy G_f_. In order to investigate the effects of interfacial strength and fracture energy, parametric studies of five different material inputs for σ* and G_f_ were carried out. These two parameters for the interfacial transition zone (itz) are varied relative to the properties of the concrete for each simulation while the other parameters were constant; i.e., they are ranging from 10% (σ* = 0.6 MPa, G_f_ = 0.006 N/mm) to 100% (σ* = 6 MPa, G_f_ = 0.06 N/mm).

## 5. Results and Discussion

The effects of the interfacial cohesive properties, the microcapsule size, and volume fraction on the load carrying capacity and the crack pattern of self-healing concrete are examined in this section by using the results of the numerical simulations. In addition the obtained results will be compared with other results from previous computational fracture modeling of encapsulated self-healing concrete in order to validate the developed design method of microcapsules.

### 5.1. Effects of Interfacial Fracture Properties and the Microcapsule Size on the Load Carrying Capacity

**Figure 14:**
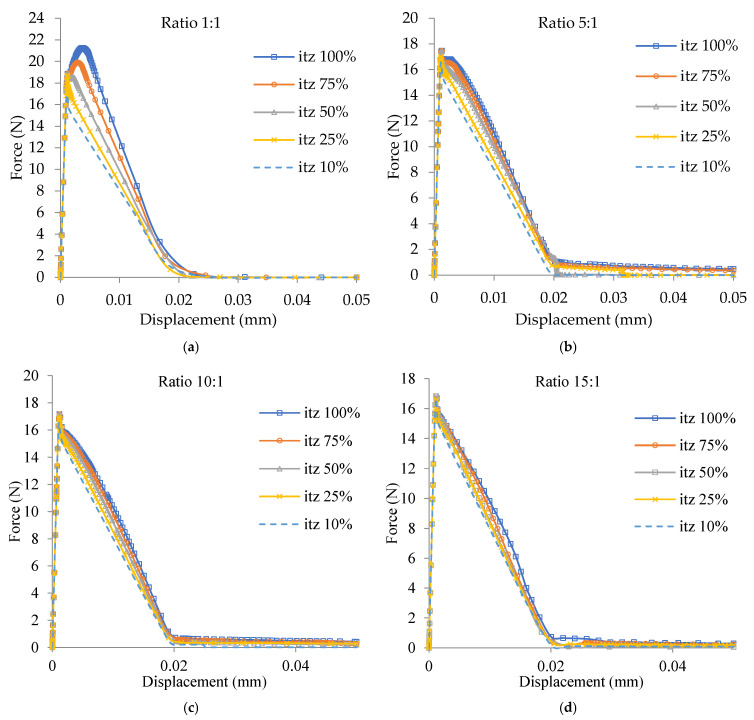
Load displacement curves for microcapsule *V_f_* = 5% with different itz values and four core–shell ratios: (**a**) 1:1; (**b**) 5:1; (**c**) 10:1; (**d**) 15:1.

**Figure 15:**
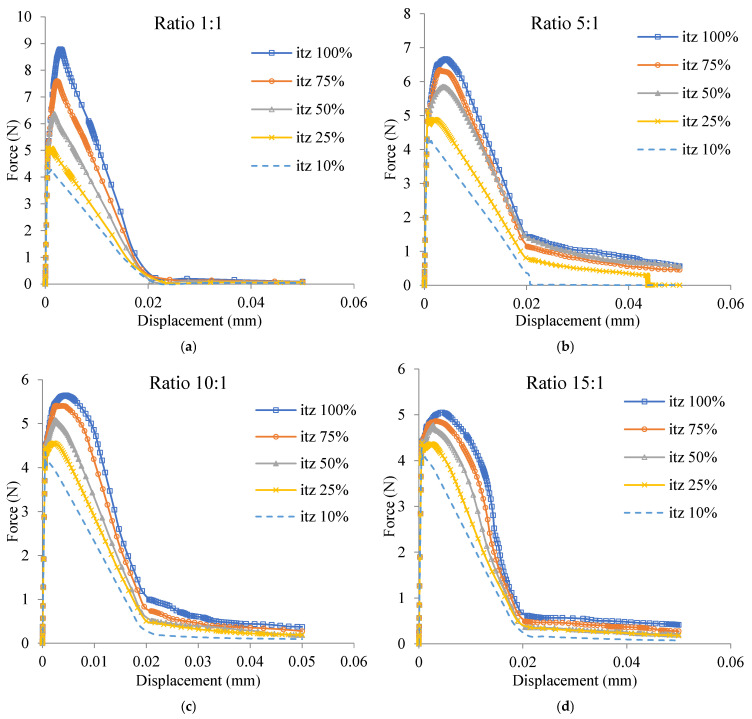
Load displacement curves for microcapsule *V_f_* = 25% with different itz values and four core–shell ratios: (**a**) 1:1; (**b**) 5:1; (**c**) 10:1; (**d**) 15:1.

The effects of interfacial fracture properties (itz) for the design samples with core–shell ratios 1:1, 5:1, 10:1, and 15:1 on load carrying capacity are defined by variation of itz from 10% of the concrete fracture properties (σ* = 0.6 MPa, G_f_ = 0.006 N/mm) to 100%; this is the same as the concrete fracture properties (σ* = 6 MPa, G_f_ = 0.06 N/mm), which are demonstrated in Figure 14a–d for *V_f_* = 5% and in Figure 15a–d for *V_f_* = 25%. Figure 14a shows the effects of the interfacial fracture properties (itz) on the load carrying capacity of the design sample for the microcapsule *V_f_* = 5% with core–shell ratio 1:1. The maximum load of the design sample decreased from 21.3 N for itz = 100% to 16.9 N for itz = 10%. Figure 15 shows the effects of the interfacial fracture properties (itz) on the load carrying capacity of the design sample for the microcapsule *V_f_* = 25% with core–shell ratio 1:1. The maximum load of the design sample decreased from 8.9 N for itz = 100% to 4.5 N for itz = 10%. As a result, it is clear that the interfacial cohesive properties (itz) have a significant role in governing the load carrying capacity of the design samples. The same phenomenon can also be found in Figure 14b–d for the microcapsule *V_f_* = 5% and in Figure 15b–d for the microcapsule *V_f_* = 25% with ratios 5:1, 10:1, and 15:1, respectively. It is clear that when the interfacial cohesive fracture properties have the same values of the concrete matrix, the higher maximum load carrying capacity will be achieved. Consequently, it is obvious that the higher the itz, the higher the load carrying capacity and vice versa. These results are in good agreement with the results obtained from previous computational fracture modeling of encapsulated self-healing concrete [10,14].

### 5.2. Effects of Interfacial Fracture Properties and the Microcapsule Size on the Maximum Carrying Load

**Figure 16:**
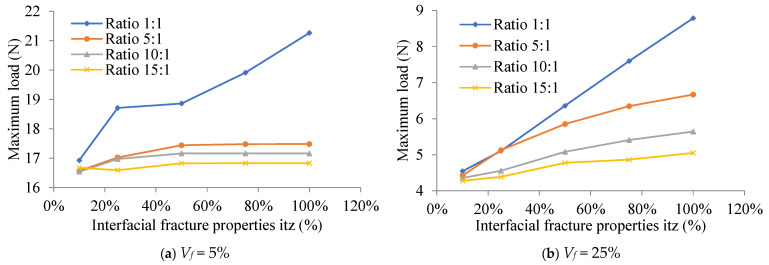
Effects of interfacial fracture properties and the microcapsule size on the maximum carrying load for *V_f_* = 5%.

The core–shell ratio is used to define the effects of the microcapsule size on the maximum carrying load for the four design samples with different interfacial fracture properties (itz), which are shown in Figure 16. It is demonstrated that the sample’s maximum carrying load increases with decreasing core–shell ratio (increasing shell thickness) in the microcapsule. Conversely, a higher itz value increases the maximum load that the design sample is capable of carrying. It also shows that the varying effect of itz on the microcapsule *V_f_* = 5% with lower shell thickness is almost constant, as seen in Figure 16a for microcapsules core–shell ratios 10:1 and 15:1.

### 5.3. Effects of Interfacial Fracture Properties and the Microcapsule Size on the Crack Pattern

**Figure 17:**
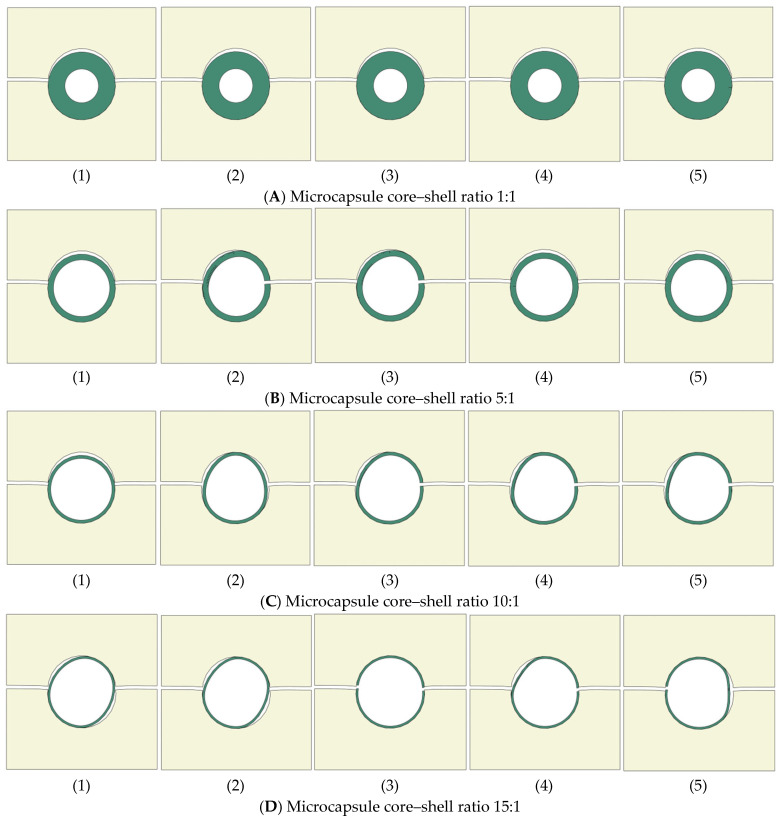
Crack pattern of microcapsule *V_f_* = 5% with four core–shell ratios and different itz values: (1) itz = 10%, (2) itz = 25%, (3) itz = 50%, (4) itz = 75%, (5) itz = 100%.

**Figure 18:**
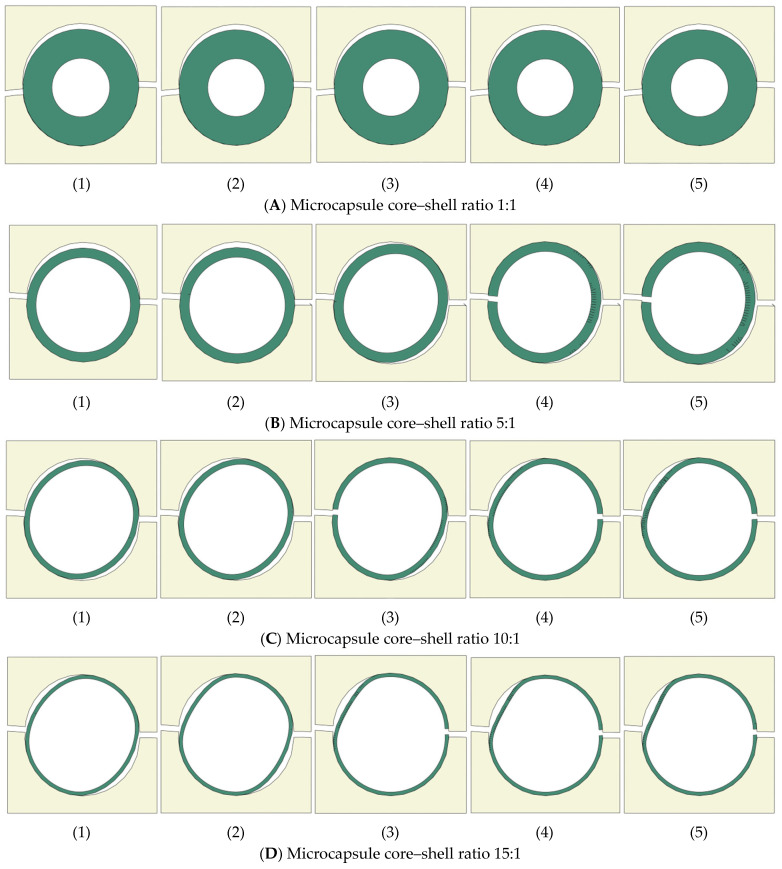
Crack pattern of microcapsule *V_f_* = 25% with four core–shell ratios and different itz values: (1) itz = 10%, (2) itz = 25%, (3) itz = 50%, (4) itz = 75%, (5) itz = 100%.

The effects of the interfacial fracture properties (itz) and microcapsule core–shell ratios 1:1, 5:1, 10:1, and 15:1 on the crack pattern are shown in Figure 17A–D for the design samples with volume fraction *V_f_* = 5% and in Figure 18A–D for *V_f_* = 25% respectively. The effects of the interfacial fracture properties are represented by variation of itz from 10% of the concrete fracture properties (σ* = 0.6 MPa, G_f_ = 0.006 N/mm) to 100%; this is the same as the concrete fracture properties (σ* = 6 MPa, G_f_ = 0.06 N/mm). Figure 17A illustrates that design samples with *V_f_* = 5% and microcapsule core–shell ratio 1:1 (the largest shell thickness) produced the same crack patterns as the microcapsule that is debonded from the concrete matrix, regardless of the ratio of the interfacial fracture properties (itz). The propagating crack could not break the microcapsule shell even when the interfacial fracture properties have the same values of the concrete matrix—see Figure 17A(1–5)—as an interfacial crack occurs regardless the itz ratio. The same phenomena happened for design samples with *V_f_* = 25% and microcapsule core–shell ratio 1:1—see Figure 18A. Figure 17B illustrates that for design samples with *V_f_* = 5%, microcapsule core–shell ratio 5:1, and when the itz ratio of the concrete fracture properties ranging between 0% and 50%, an interfacial crack occurred, and the microcapsule is debonded from the concrete matrix, as shown in Figure 17B(1–3). When the itz is ranged between 75 and 100%, the propagating crack became an interfacial crack when it reached the microcapsule shell and broke it from the other side, as illustrated in Figure 17B(4,5). That means that a partial fracture crack is developed when the microcapsule core–shell ratio is 5:1 and the ratio of the interfacial fracture properties between the microcapsule and the concrete matrix (itz) is ranging between 75 and 100%. The same phenomena happened for the design samples with *V_f_* = 25% and microcapsule core–shell ratio 5:1—see Figure 18B. Figure 17C illustrates the fracture pattern for the design samples with *V_f_* = 5% and microcapsule core–shell ratio 10:1 and varying itz ratios. When itz range between 0% and 25%, an interfacial crack occurs and the microcapsule is debonded from the concrete matrix as shown in Figure 17C(1,2). However, when the itz ranges between 50 and 100%, the propagating crack became an interfacial crack when it reached the microcapsule shell and broke it from the other side, as illustrated in Figure 17C(3–5). The same phenomena happened for the design samples with *V_f_* = 25% and microcapsule core–shell ratio 10:1—see Figure 18C. Figure 17D illustrates the fracture pattern for the design samples with *V_f_* = 5% and microcapsule core–shell ratio 15:1 and varying itz ratios. When itz ranges between 0% and 25%, interfacial cracks occurred, and the microcapsule is debonded from the concrete matrix, as shown in Figure 17D(1,2). However, when the itz ranges between 50 and 100% the propagating crack could break the microcapsule, as illustrated in Figure 17D(3–5). The same phenomena happened for the design samples with *V_f_* = 25% and microcapsule core–shell ratio 15:1—see Figure 18D. These results are in good agreement with the results obtained from previous computational fracture modeling of encapsulated self-healing concrete [10,14].

It can be easily noticed by comparing the crack patterns generated from the design samples with volume fraction (*V_f_* = 5%) in Figure 17 and the crack patterns generated from the design samples with volume fraction (*V_f_* = 25%) in Figure 18 that increasing the volume fraction leads to an increase in the possibility of the microcapsule being fractured.

## 6. Conclusions

A simple design method of dimensioning microcapsules for encapsulation-based self-healing concrete (SHC) with consideration for a sufficient volume of healing agent to heal a specific crack width is developed in this paper. The developed design method is based on the configuration of the representative volume element (RVE), the unit cell (UC), and associates them with the volume fraction (*V_f_*) and the crack width as variables with periodic boundary conditions (PBCs) applied. The developed design method is also validated through numerical fracture modeling using the extended finite element method (XFEM), and cohesive surface (CS) technique. The effects of microcapsule size and volume fraction (*V_f_*) on load carrying capacity and the likelihood of microcapsule fracture or debonding from the concrete matrix are investigated numerically in this validation. 2D numerical fracture modeling is performed for the developed SHC design samples including the designed microcapsules, which loaded under uniaxial tension with PBCs applied. The concrete matrix and the microcapsule shell are modeled using XFEM, while CS models the contact surface between them. The following conclusions can be made:

- The obtained results from the numerical simulations for the developed design of microcapsules are in good agreement with the results obtained from previous computational fracture modeling of encapsulated self-healing concrete.
- The interfacial cohesive characteristics have a significant impact on the load carrying capacity of the design samples as the higher the itz, the higher the load carrying capacity and vice versa.
- The microcapsule size represented the by core–shell ratio has an impact on the maximum carrying load of the design samples. The maximum carrying load of the sample increases with decreasing the core–shell ratio (increasing shell thickness) of the microcapsule.
- The interfacial cohesive characteristics (itz) are directly proportional to the design sample strength. On the contrary, the microcapsule core–shell ratio and the volume fraction (*V_f_*) are inversely proportional to the sample strength. It has also been noticed that the varying of the itz on the microcapsule with higher microcapsule core–shell ratio and lower *V_f_* almost has no significant effect on the design sample strength.
- Increasing the volume fraction (*V_f_*) leads to an increase in the possibility of the microcapsule being fractured.
- The interfacial fracture properties (itz), the microcapsule core–shell ratio, and the volume fraction (*V_f_*) also affect the crack pattern as increasing them leads to an increase in the possibility of the microcapsule being fractured. Though, the microcapsule core–shell ratio of 1:1, which has the thickest shell but the smallest healing agent volume, is not advised. As a result, irrespective of the itz ratios, the microcapsule will debond from the concrete. The likelihood of the microcapsule debonding from the concrete increases generally when the itz is less than 50% of concrete fracture properties. A partial fracture/interfacial crack develops when the itz ranges between 50 and 75%. The microcapsule fractures when the itz ranges between 75 and 100%.
- The developed design method for microcapsules is very simple and neglects the random distribution of the microcapsules. However, it is a helpful tool to design the microcapsule size with the consideration of the sufficient volume of healing agent to heal a specific crack width. This design method can help practical engineers to obtain a preliminary design of microcapsules for self-healing concrete without any complications, especially for costly detailed modeling.

Future works for this developed microcapsule design method could study more complex models such as fracture modeling of microcapsules designed by this method and distributed randomly inside the sample to mimic the realities of SHC members. The Monte Carlo simulation should be applied to generate such random distribution of the microcapsules. However, these simulations would be computationally expensive in addition to the complications generated from mesh discretization and capsular clustering. Computational simulations for RVE samples could be a useful improvement to reduce the computational cost by studying the proper representing RVE size. The first step in carrying out these investigations is to look at XFEM’s limitations, which primarily allow for the creation of several fractures within the same enrichment zone. These limitations may be addressed by adjusting the Abaqus input file. 3D computational simulations should also be considered for future work to validate the 2D computational simulation and estimate the accuracy difference ratio between 2D and 3D simulations, which allows practical engineers to get much more reliable results based only on 2D simulations that can be built easily and are not as computationally expensive as 3D simulations.

## Figures and Tables

**Table 1:** The material properties.

Material	E (MPa)	ν	σ* (MPa)	G_f_ (N/mm)
Concrete	25,000	0.2	6	0.06
Capsule	3600	0.3	10	0.1
Interface	-	-	Varies	Varies

## Data Availability

The original contributions presented in this study are included in the article. Further inquiries can be directed to the corresponding author.
